# Best Supportive Care *Versus* Whole-Brain Irradiation, Chemotherapy Alone, or WBRT Plus Chemotherapy in Patients With Brain Metastases From Small-Cell Lung Cancer: A Case-Controlled Analysis

**DOI:** 10.3389/fonc.2021.568568

**Published:** 2021-03-01

**Authors:** Hongwei Li, Ruiqi Xue, Xiaotang Yang, Songye Han, Weihua Yang, Xin Song, Xiaqin Zhang, Jianzhong Cao, Sufang Jia, Weili Wang, Jianhong Lian

**Affiliations:** ^1^ Department of Radiotherapy, Shanxi Medical University, Shanxi Cancer Hospital, Taiyuan, China; ^2^ Department of Radiology, Shanxi Cancer Hospital, Taiyuan, China; ^3^ Department of Chemotherapy, Shanxi Medical University, Shanxi Cancer Hospital, Taiyuan, China; ^4^ Department of Surgery, Shanxi Medical University, Shanxi Cancer Hospital, Taiyuan, China

**Keywords:** small cell lung cancer, brain metastases, whole-brain irradiation, chemotherapy, best supportive care

## Abstract

**Background:**

WBRT and systemic chemotherapy are the mainstay treatments for small-cell lung cancer (SCLC) brain metastases (BM). However, current recommendations are mainly based on evidence from retrospective analyses. A recent randomized trial found no benefits from WBRT compared with best supportive care (BSC) in patients with more than three BM from non-small-cell lung cancer (NSCLC). Herein, we aimed to evaluate the roles of WBRT and chemotherapy further in the management of BM from SCLC.

**Materials and Methods:**

There were 698 patients with BM from SCLC included. Of these, 580 received anti cancer treatment (Group 1), including 178 who received WBRT only (Group 1a), 129 who received chemotherapy only (Group 1b), and 273 who received WBRT plus chemotherapy (Group 1c). The other 118 received BSC (Group 2). Propensity score matching (PSM) analysis was used to compare Group 2 with each of the other groups.

**Results:**

After PSM, compared with Group 2 (n = 118), patients in Group 1 (n = 440) had a prolonged overall survival (OS) in both univariate and multivariate tests, with a median survival time of 10 months (95% CI = 9−11) in Group 1 and 3.5 months (95% CI = 2−7) in Group 2 (p < 0.001). In subgroup analyses, patients who received WBRT plus chemotherapy were more likely to benefit from treatment (p < 0.001). Chemotherapy alone or WBRT alone did not show survival benefits.

**Conclusion:**

WBRT plus chemotherapy improved OS in patients with BM from SCLC as compared to BSC. Chemotherapy alone and WBRT alone did not show survival benefits. This retrospective study suggests that SCLC patients with BM who receive WBRT combined with chemotherapy have a better outcome than those receiving BSC alone.

## Introduction

SCLC constitutes about 15% of all lung cancer cases (1). Approximately two-thirds of SCLC patients present with metastatic disease. Thus SCLC has a dismal prognosis with a median survival of less than 10 months (2). The central nervous system is the most frequent metastatic site among SCLC patients, accounting for 10–15% of the cases at diagnosis. Furthermore, approximately 80% of SCLC patients develop BM in the process of the whole disease (3, 4). Currently, WBRT is the mainstay treatment for SCLC BM regardless of the number of intracranial metastases, with a recommended dose of 30 Gy in 10 fractions or 40 Gy in 20 fractions (5). The main reason for the use of WBRT is that SCLC patients tend to develop multiple BM (6, 7). With WBRT, the median survival time can improve to 4 to 6 months from 1 to 2 months, and 50−60% of the patients have Central Nervous System (CNS) symptom remission (8, 9). Systemic chemotherapy has also been suggested as an important palliative modality due to the general belief that SCLC is the most aggressive type of lung cancer and is chemotherapy-sensitive. However, these recommendations are mainly based on evidence from retrospective analyses over the last three decades (10, 11). With improvements in systemic therapy and prolonged survival in this kind of patients, the observed toxicities, especially cognitive deterioration after WBRT, have been the main focus (12, 13). A recent randomized trial (the QUARTZ study) also found no benefit from WBRT compared with BSC in BM patients with NSCLC (14). These results have raised questions regarding the use of WBRT and/or chemotherapy in patients with BM from SCLC. To define the optimal treatment in the management of BM from SCLC better, we performed a retrospective analysis based on PSM analysis using real-world data to evaluate whether WBRT, chemotherapy, or WBRT plus chemotherapy has survival benefits in patients with BM from SCLC compared with BSC.

## Materials and Methods

### Patient Eligibility

We reviewed the records of patients with BM from 3,651 SCLC cases treated between May 2004 and August 2017 at Shanxi Provincial Cancer Hospital in Taiyuan, China. All of the patients were diagnosed by pathologic examination. BM were confirmed by contrast-enhanced computed tomography (CT) or gadolinium-enhanced magnetic resonance imaging (MRI) at the initial diagnosis or during the progression of the disease.

**Figure 1:**
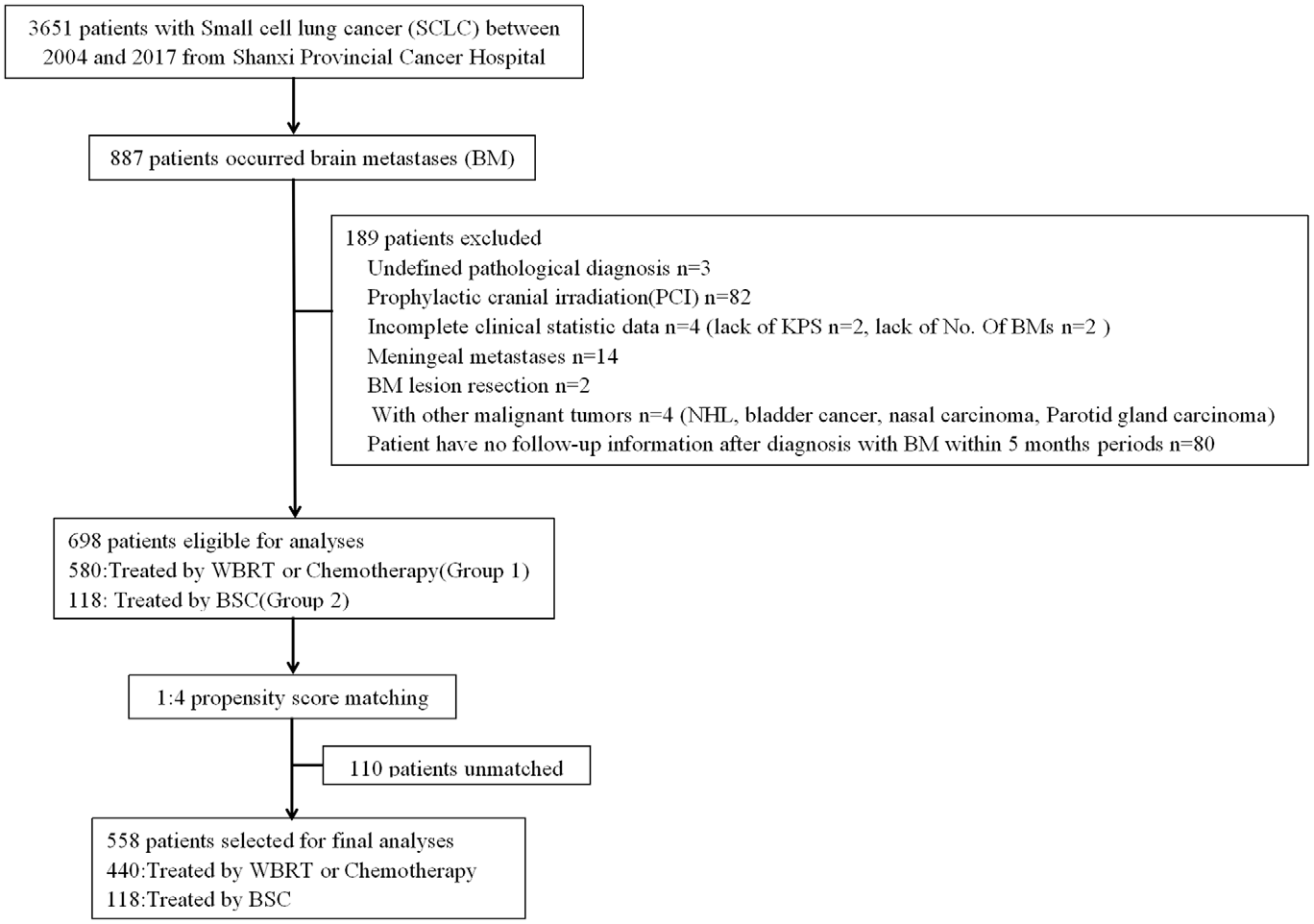
work flow diagram of patients selection.

Patients who met the following criteria were excluded: (1) history of other malignant tumors; (2) received prophylactic cranial irradiation (PCI); (3) incomplete demographic, clinical, and/or outcome data; (4) meningeal metastases; and (5) Patient has no follow-up information after diagnosis with BM within 5 months periods. Consequently, 698 patients were included in the present study (**Figure 1**). Patient-related variables included gender, age (≤65 *vs.* >65), Karnofsky Performance Status score (KPS) (≥90 *vs.* 70−80 *vs.* ≤60), history of smoking (yes *vs.* no), extracranial systemic metastases (present *vs.* absent), number of BM (1 *vs.* 2−3 *vs.* >3), and time of metastases (synchronous *vs.* metachronous). Synchronous metastases were defined as brain lesions were discovered at the time of initial diagnosis of the primary tumor or within 1 month thereafter and without receiving anticancer treatments. All others were defined as metachronous especially in the process of diseases. Treatment options included WBRT, chemotherapy, a combination treatment of these two options, and BSC. Radiotherapy was applied with two lateral fields with a 6-MV linear accelerator or conformal radiation therapy (CRT)/intensity modulated radiation therapy (IMRT). The portal included the whole brain with inclusion of the skull-based areas. The total dose was 30 Gy in 10 fractions or 40 Gy in 20 fractions. The majority of chemotherapy regimens were etoposide/carboplatin, etoposide/cisplatin, or oral etoposide for those with lower health status. Patients who received more than one cycle were classified as having chemotherapy. BSC included steroid or other supportive care. All of the cases based on treatment modality were categorized in groups: patients who received WBRT, chemotherapy, or a combination of two modalities (Group 1), and those who received BSC (Group 2). Among Group 1, chemotherapy was defined as Group 1a; patients who received WBRT were defined as Group 1b; patients who received a combination of the two modalities were defined as Group 1c. To define optimal treatment in the management of BM from SCLC, we compared the survival benefit between patients receiving anticancer treatment (including WBRT, chemotherapy, or a combination of the two modalities) (Group 1) and those who received only BSC (Group 2). The survival differences between subgroups WBRT only (Group 1a) *vs.* BSC (Group 2), chemotherapy only (Group 1b) *vs.* BSC (Group 2), and a combination of WBRT and chemotherapy (Group 1c) *vs.* BSC (Group 2) were further analyzed.

The Institutional Review Board of Shanxi Cancer Hospital approved the study and the informed consent waiver.

### Statistical Analysis

The primary endpoint of this study was OS, defined from the date of BM diagnosis to death or last follow-up. OS and prognostic factors were evaluated in univariate and multivariate analyses. Differences between the systems and their predictive values were evaluated. Either the Chi-squared or the Fisher exact test was used to compare the proportion between two groups. OS was estimated using the Kaplan-Meier method. Group analysis was performed using the log-rank test for univariate analyses and the Cox proportional hazards model for multivariate analyses. To minimize confounding effects due to the non-randomized assignment, PSM was used. A propensity score for each patient was calculated by logistic regression using the factors of age, gender, smoking, KPS, brain metastatic numbers, extracranial status, and time of metastases. One 1:4 (Group 2 *vs.* Group 1) and three 1:1 matched groups (Group 2 *vs.* Group 1a, Group 2 *vs.* Group 1b, and Group 2 *vs.* Group 1c) were created using the nearest neighbor matching algorithm. The caliper size was calculated as 0.20 * standard deviation of the propensity score. The robustness of the propensity score distributions was evaluated graphically, and the balance was evaluated by computing the standardized difference of the covariates across the two groups. P < 0.05 was considered significant. All of the analyses were performed using R version 2.15.2 (15).

## Results

Of the 698 patients with BM included in this analysis, 58 patients were confirmed by CT, the other 640 patients by MRI. Five hundred eighty received anticancer treatments (Group 1), including 178 treated with WBRT (Group 1a), 129 with chemotherapy (Group 1b), and 273 with a combination of WBRT and chemotherapy concurrently or sequentially (Group 1c). The other 118 patients only received BSC (Group 2). The median follow-up time for all of the patients was 7 months. In total, the 1-year survival rates and the median survival time were 38.5% and 9 months, respectively.

After PSM analysis, 558 patients were available for PSM balance check and distribution between Groups 2 and 1 (**Supplement 1 Table A**). There were 118:440 well-balanced pairs of patients used for outcome comparison of Group 2 *vs.* Group 1. The clinical characteristics between the two groups before and after PSM are summarized in **Table 1**. In the matched patients, no differences between Groups 1 and 2 were observed for gender (p = 0.912), age (p = 0.789), smoking (p = 0.882), KPS (p = 0.453), time of metastases (p = 0.877), number of BM (p = 0.977), or ECM (p = 0.921).

**Table 1:** Comparison of 698 Patients Characteristics before and after matching.

Characteristics	Х^2^		Х^2^ after PSM	
	Group 1 Group 2	*P*	Group 1 Group 2	*P*
Total	580 (83.1%) 118 (16.9%)		440 (78.9%) 118 (21.1%)	
Gender Male Female	105 (18.1%) 19 (15.3%) 475 (81.9%) 99 (17.2%)	0.604	69 (15.7%) 19 (16.1%) 371 (84.3%) 99 (83.9%)	0.912
Age <65 ≥65	426 (73.4%) 83 (70.3%) 154 (26.6%) 35 (29.7%)	0.488	315 (71.6%) 83 (70.3%) 125 (28.4%) 35 (29.7%)	0.789
Smoking No Yes	150 (25.9%) 25 (21.2%) 430 (74.1%) 93 (78.8%)	0.285	96 (21.8%) 25 (21.2%) 344 (78.2%) 93 (78.8%)	0.882
KPS (Wilcoxon) ≤60 70–80 ≥90	75 (12.9%) 19 (16.1%) 282 (48.6%) 60 (50.8%) 223 (38.5%) 39 (33.1%)	0.210	61 (13.9%) 19 (16.1%) 220 (50.0%) 60 (50.8%) 159 (36.1%) 39 (33.1%)	0.453
Timing of BM Metachronous Synchronous	178 (31.7%) 34 (28.8%) 402 (69.3%) 84 (71.2%)	0.686	130 (29.5%) 34 (28.8%) 310 (70.5%) 84 (71.2%)	0.877
No. of BMs (Wilcoxon) 1 2–3 >3	232 (40.0%) 48 (40.7%) 145 (25.0%) 25 (21.2%) 203 (35.0%) 45 (38.1%)	0.792	179 (40.7%) 48 (40.7%) 92 (20.9%) 25 (21.2%) 169 (38.4%) 45 (38.1%)	0.977
Extracranial Disease Control Yes No	354 (61.0%) 73 (61.9%) 226 (39.0%0 45 (38.1%)	0.866	270 (61.4%) 73 (61.9%) 170 (38.6%) 45 (38.1%)	0.921

Group 1, Treated by WBRT or Chemotherapy; Group 2, Treated by BSC; PSM, Propensity Score Matching; BM, Brain Metastases; KPS, Karnofsky Performance Status score; GPA, Graded Prognostic Assessment; WBRT, Whole Brain Radiotherapy. Percentages are in brackets.

**Figure 2:**
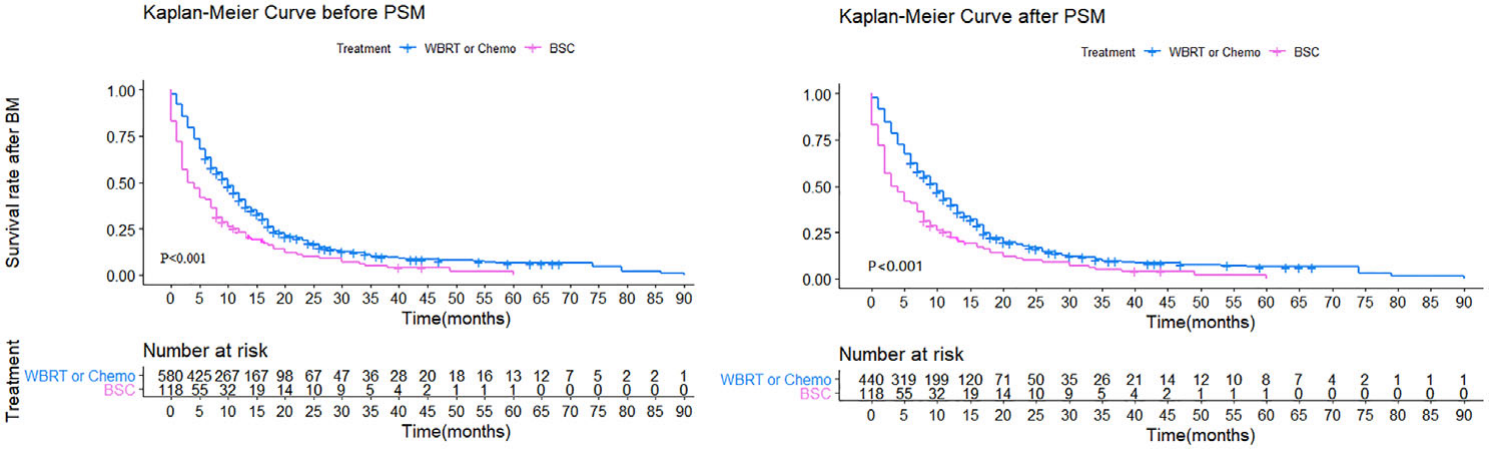
Kaplan-Meier survival curves showing WBRT or Chemotherapy (Group 1) and BSC (Group 2).

For naive unmatched patients, the median survival time was 10 months (95% CI = 9−11) in Group 1 *vs.* 3.5 months (95% CI = 2−7) in Group 2 (p < 0.001). The 1-year survival rates for patients in Groups 1 and 2 were 40.8 and 28.0%, respectively (p < 0.001). After PSM, the median survival time was 10 months (95% CI = 9−11) in Group 1 *vs.* 3.5 months in Group 2 (95% CI = 2−7) (p < 0.001). The 1-year survival rates for patients in Groups 1 and 2 were 36.4 and 22.0%, respectively (p < 0.001). To reduce the effect of confounding factors in this retrospective study, Cox proportional hazards regression analyses were used in both the matched and unmatched patients. In the naive unmatched patients, the independent factors for better survival were higher KPS score, lower number of BM, absence of extracranial metastases, and treatment. The matched patients showed similar results. Both in the matched and unmatched patients, those in Group 1 showed survival superiority compared to Group 2 (p < 0.001) (**Figure 2**, **Table 2**).

**Table 2:** Univariate and Mutivariate of cox analysis of 698 patients before and after PSM.

Characteristics	Before PSM								After PSM							
	Log-rank				COX				Log-rank				COX			
	n/events	MST(m)	95% CI	*P*	HR	Z(Wald)	*P*	*ZPH*	n/events	MST(m)	95% CI	*P*	HR	Z(Wald)	*P*	*ZPH*
Gender Male Female	124/100 574/493	11 8	9–15 7–10	0.009	0.163	0.972	0.331	0.082	88/70 470/405	10 8	8–13 7–10	0.1	—	—	—	—
Age <65 ≥65	509/420 189/173	9 8	8–10 6–10	0.02	1.182	1.828	0.068	0.15	398/329 160/146	9 7	8–10 5–10	0.02	1.197	1.793	0.073	0.179
Smoking No Yes	175/140 523/453	11 8	9–15 7–10	0.003	1.108	0.710	0.477	0.418	121/97 437/378	10 8	7–13 7–9	0.1	—	—	—	—
KPS ≤60 70–80 ≥90	94/82 342/302 262/209	6 9 11	4–11 8–10 8–13	<0.001	0.765 0.597	−2.114 −3.843	0.035 <0.001	0.855 0.058	80/71 280/247 198/157	6 8 10	4–11 7–10 8–13	<0.001	0.774	−1.877	0.061 <0.001	0.631 0.066
BM Time Metachronous Synchronous	212/180 486/413	9 9	8–11 7–10	0.08	—	—	—	—	164/139 394/336	9 8	7–11 7–10	0.1	—	—	—	—
No. of BMs 1 2–3 >3	280/254 170/137 248/202	6 10 12	5–7 9–12 11–14	<0.001	0.662 0.539	−3.868 −6.351	<0.001 <0.001	0.123 0.081	227/208 117/96 214/214	6 10 12	5–7 8–12 10–14	<0.001	0.683 0.530	−3.07 −5.95	0.002 <0.001	0.375 0.407
Extracranial Control Yes No	427/372 271/221	7 12	6–8 10–13	<0.001	0.713	−3.912	<0.001	0.252	343/300 215/175	7 11	6–8 10–13	<0.001	0.732	−3.232	0.001	0.035
Treatment Group 1 Group 2	580/482 118/111	10 3.5	9–11 2–7	<0.001	1.843	5.692	<0.001	0.058	440/364 118/111	10 3.5	9–11 2–7	<0.001	1.807	5.377	<0.001	0.061

**Figure 3:**
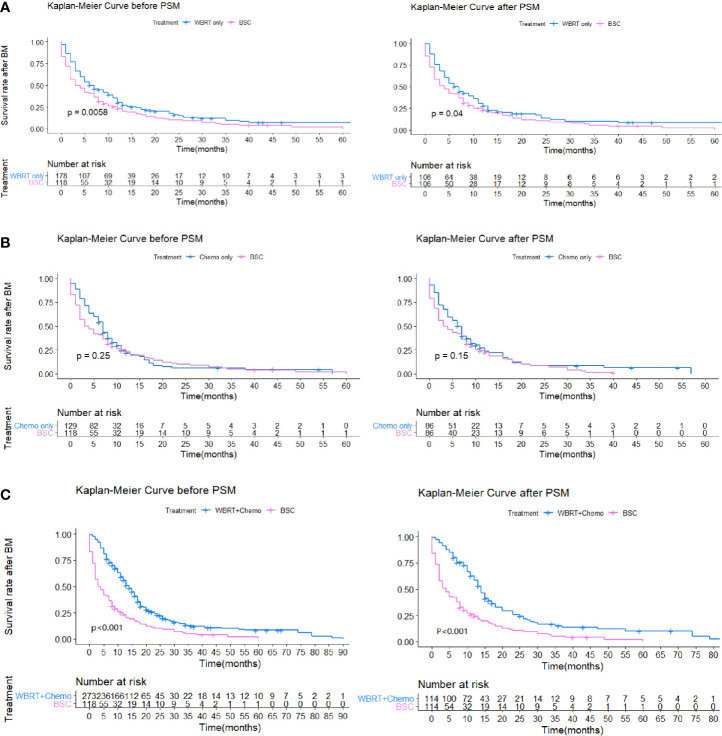
**(A)** Kaplan-Meier survival curves showing WBRT only (Group 1a) and BSC (Group 2). **(B)** Kaplan-Meier survival curves showing Chemotherapy only (Group 1b) and BSC (Group 2). **(C)** Kaplan-Meier survival curves showing WBRT combined with Chemotherapy (Group 1c) and BSC (Group 2).

To identify which specific treatment modality would have survival benefits when comparing with patients who received only BSC, subgroup survival analyses for Group 2 *vs.* Group 1a, Group 2 *vs.* Group 1b, and Group 2 *vs.* Group 1c were also conducted based on PSM. Then, 1:1 PSM was performed in each comparative group. PSM balance check and distribution between groups are presented in **Supplement 1 Table B–D** and **Supplement 2 Tables A–C**. All three comparative groups were well matched. After PSM, both in univariable and multivariable analyses, the results showed that patients in Group 1b had no statistically significant survival benefits over those in Group 2 (p = 0.2). Those in Group 1a showed statistical differences compared to Group 2 by a log-rank test (p = 0.04). However, the Cox regression model demonstrated no survival superiority (p = 0.22). For patients in Group 1c, we found that WBRT plus chemotherapy had survival superiority over Group 2 both in univariable (p < 0.001) and multivariable analyses (p < 0.001) (**Table 3**, **Figure 3**).

**Table 3:** Summary of the Univariate and Mutivariate analysis results of different treatments before and after PSM.

Characteristics	Before PSM								After PSM							
	Log-rank				COX				Log-rank				COX			
	n/events	MST(m)	95% CI	*P*	HR	Z(Wald)	*P*	*ZPH*	n/events	MST(m)	95% CI	*P*	HR	Z(Wald)	*P*	*ZPH*
Treatment Group 1 Group 2	580/482 118/111	10 3.5	9–11 2–7	<0.001	1.843	5.692	<0.001	0.058	440/364 118/111	10 3.5	9–11 2–7	<0.001	1.807	5.377	<0.001	0.061
Treatment Group 1a Group 2	178/150 118/111	6.5 3.5	5–9 2–7	0.006	1.474	3.006	0.003	0.051	106/89 106/99	6.5 4.0	5–9 2–7	0.04	1.4007	2.292	0.22	0.169
Treatment Group 1b Group 2	129/110 118/111	7.0 3.5	6–8 2–7	0.25	—	—	—	—	86/74 86/81	6.5 3.5	4–8 2–7	0.15	—	—	—	—
Treatment Group 1c Group 2	273/222 118/111	14.0 3.5	13–16 2–7	<0.001	2.428	7.286	<0.001	0.062	114/89 114/107	14 4	12–16 2–7	<0.001	2.5343	6.242	<0.001	0.273

Group 1, Treated by WBRT or Chemotherapy; Group1a, WBRT only; Group1b, Chemotherapy only; Group 2, Treated by BSC;Group 1c:WBRT combined with Chemotherapy; MST, Medium Survival Time; SE, Stand Error; CI, Confidence Interval; HR, Hazard Ratio; PSM, Propensity Score Matching; BM, Brain Metastases; KPS, Karnofsky Performance Status score.

## Discussion

The results of this retrospective case-controlled study of 698 patients revealed that anticancer treatment was associated with improved OS for SCLC patients with BM compared to those with only BSC. Among patients receiving anticancer treatment, those receiving WBRT plus chemotherapy were more likely to benefit from treatment. Neither chemotherapy nor WBRT alone showed survival benefits.

BM presents a dismal prognosis in patients with SCLC, with a median survival between 2 and 14 months (16). WBRT and palliative chemotherapy are currently recommended as the standardized treatment modality due to the frequent occurrence of multiple metastases (17, 18). However, evidence has mainly been acquired in small retrospective and nonrandomized trials. Only one small size randomized study from 48 patients by the Eastern Cooperative Oncology Group (ECOG) confirmed that WBRT plus supportive care showed survival benefit in its secondary end point (19). Due to the limited number of subjects, in previous studies, SCLC BM patients have always been grouped with BM from other solid tumors, especially with NSCLC (10, 11). Recently, a phase III randomized trial by Mulvenna et al. in 2016 (the QUARTZ study) showed no benefits from WBRT as compared to BSC in BM patients from NSCLC. Only those in specific subgroup (<60 years of age, >79 KPS) had better prognosis (14). Moreover, BM patients from SCLC were prone to have advanced disease and spread from primary disease compared to patients with other solid tumors (7, 20, 21). In addition, some late complications, such as neurocognitive impairment, may occur after WBRT (12, 13). Thus, there is a need to redefine the value of WBRT specific for BM from SCLC. For treatment of systematic chemotherapy, although SCLC is a chemotherapy-sensitive cancer, it is recognized that effects on brain lesions are generally poor because the blood-brain barrier (BBB) limits most drugs’ penetration into the brain. In this study, we first compared 580 SCLC BM patients who received anticancer treatment (Group 1) including WBRT chemotherapy or a combination of these two modalities with 118 patients receiving BSC only (Group 2). The results showed that the patients in Group 1 had prolonged OS with significant statistical differences both in univariate and multivariate tests. After PSM, similar results were obtained. This might imply that positive treatment involvement could bring more survival benefit than BSC only.

To define the exact role of different treatment modalities in SCLC BM patients further, we compared the OS of patients treated with WBRT (Group 1a), chemotherapy alone (Group 1b), and WBRT plus chemotherapy (Group 1c) with BSC (Group 2), based on PSM. The results showed that WBRT alone did not show survival benefit in comparison with BSC, which was in line with the QUARTZ study. WBRT has remained standard practice for treating BM from all solid primary tumors since the 1970s. The doses and fractions from 30 Gy in 10 fractions to 40 Gy in 20 fractions provide similar survival times (18). The treatment regimen is almost the same for all kinds of BM from different malignancies. Actually, because of the extremely heterogeneous biological nature of cancers, the survival time after WBRT would not always be the same (22). SCLC is one of the most aggressive types of cancer. When patients are diagnosed with BM, most of them already have other crucial organ metastases and cancer dissemination (23). In addition, some distant metastases might exist, which are not identifiable by imaging tests (24). Therefore, due to uncontrolled cancer, although WBRT alone could deal with the cranial diseases quite effectively, a prolonged survival time may not be achieved. In the present study, our results favored the above possibilities.

The subgroup comparison of Group 1b *vs.* Group 2 showed that systemic chemotherapy did not present a survival benefit over BSC. The systemic treatment paradigms for SCLC have not been developed for 20 years. Regimens of etoposide/carboplatin and etoposide/platinum remain the first-line treatment suggestions (25). For BM, chemotherapy has not been the first line of treatment because of the notion of the blood-brain barrier (BBB) preventing penetration of anticancer drugs into the central nervous system. However, other experimental and clinical evidence has shown that the BBB might be disrupted in the region around a brain metastatic lesion when the lesion’s diameter is larger than 0.5 mm (26). In addition, certain anticancer drugs, such as irinotecan and carboplatin, appear to penetrate the BBB to some extent, consequentially exerting anticancer activity. A study in asymptomatic BM from SCLC by Seute T et al. demonstrated that combination of cyclophosphamide, doxorubicin, and etoposide showed the brain response rate was 13%, while the systemic response rate was 73%. That might imply that the BBB disruption was not good enough for penetration of anticancer drug (27). Chen et al. showed that the response rates of BM from SCLC was 65% in a Phase II Trial by using the regimen of irinotecan plus carboplatin (28). However, Reveiz L et al. have reviewed three RCTs involving 192 patients in the Cochrane Database of Systematic Reviews and demonstrated that chemotherapy does not improve specific brain PFS and OS in patients with SCLC. The available evidence for the first-line selected treatment was insufficient (29). In the present case-control study, the result was consistent with this conclusion. Comparing to BSC, chemotherapy did not have survival benefits. Recently, two randomized, open-label, phase 3 trial showed that platinum-etoposide plus programmed cell death-Ligand 1(PD-L1) inhibitor followed by maintenance PD-L1 inhibitor achieved OS benefits *vs.* chemotherapy in advance stage SCLC. However, in the subgroup of BM, it did not have survival benefit. It was probably because of the small number of this cohort of patients and was not the primary endpoint. Therefore, there are no effective systemic treatment for BM from SCLC until to now (30, 31).

Positive results were observed in the subgroup comparison of Group 1c *vs.* Group 2. Patients receiving WBRT plus chemotherapy were demonstrated to have better OS than those receiving BSC in both univariate and multivariate analyses. With this combined modality, the lesions both in cranial and extracranial areas probably were eradicated, which might contribute to prolonged survival times for this cohort of patients. This finding suggested that chemotherapy should be combined with WBRT concurrently or subsequently as the treatment modality for SCLC BM patients who can tolerate this. WBRT plus chemotherapy might be the optimal treatment method in dealing with BM from SCLC. To date, there is no prospective research comparing WBRT plus chemotherapy with BSC in SCLC BM patients. Because of the retrospective nature of this study, this evidence should be used cautiously. A prospective multi-center randomized study is needed in the future.

In the present study, we also conducted prognostic tests to define the independent factors associated with OS. In the unmatched whole group of patients, the pretreatment variables associated with poor prognosis included age >65 years old, lower KPS scores, more BM, and extracranial metastases. This is consistent with previous reports (32–34). Nonetheless, the factors of synchronous *vs.* metachronous BM and age >65 years old were not associated with the outcomes. These data are not concordant with the report from Bernhardt et al. (20).

There are limitations to this study, mainly due to its retrospective nature. First, we were unable to investigate information about intracranial progression-free survival (iPFS), systematic progression-free survival (sPFS), patterns of failure, or treatment side effects. Such information could help optimize treatments in the future and assist clinicians in selecting treatment options. Second, in the present study, there was no information about the assessment of cognitive function and quality of life (QOL). This is a great concern to WBRT. Many previous studies have confirmed cognitive decline and the deterioration of QOL after receiving WBRT (35, 36). Third, although a PSM was used to minimize the confounding effect in this study, some intrinsic unmeasured confounders of this retrospective study cannot be avoided.

In conclusion, this analysis revealed that WBRT plus chemotherapy improved OS in patients with BM from SCLC as compared to treatment with BSC. Neither chemotherapy nor WBRT alone showed survival benefit. However, because of the retrospective nature of this study, these findings should be interpreted with caution. Future multicenter randomized controlled trials are needed to confirm our results further.

## Data Availability Statement

The original contributions presented in the study are included in the article/**Supplementary Material**. Further inquiries can be directed to the corresponding author.

## Ethics Statement

The Institutional Review Board of Shanxi Cancer Hospital approved the study.

## Author Contributions

XY designed this study. RX, SH, WY, XS, XZ, and JL collected the data. HL, JC, SJ, and WW did the statistics. All authors contributed to the article and approved the submitted version.

## Funding

Natural Science Foundation of Shanxi Province in China (201701D12116).

## Conflict of Interest

The authors declare that the research was conducted in the absence of any commercial or financial relationships that could be construed as a potential conflict of interest.
