# Supplementary material for: Best Supportive Care Versus Whole-Brain Irradiation, Chemotherapy Alone, or WBRT Plus Chemotherapy in Patients With Brain Metastases From Small-Cell Lung Cancer: A Case-Controlled Analysis
Source: Front Oncol. 2021 Mar 1;11:568568. doi: 10.3389/fonc.2021.568568 (PMC7957068; doi:10.3389/fonc.2021.568568)

Supplement 1. Summary of the balance results of different treatments by PSM

(1) Balance result of WBRT or Chemotherapy (Group 1) vs. BSC (Group 2) by PSM

| Group1:group2 | Summary of balance for all data(before PSM) 580:118 | Summary of balance for matched data(after PSM)440:118 |
| --- | --- | --- |
|  | Means Treated Means Control SD Control Mean Diff eQQ Med eQQ Mean eQQ Max | Means Treated Means Control SD Control Mean Diff eQQ Med eQQ Mean eQQ Max |
| distance | 0.1752 0.1678 0.0318 0.0074 0.0084 0.0082 0.0265 | 0.1752 0.1748 0.0325 0.0004 0.0025 0.0031 0.0176 |
| gender | 0.8390 0.8190 0.3854 0.0200 0.0000 0.0254 1.0000 | 0.8390 0.8425 0.3647 -0.0035 0.0000 0.0000 0.0000 |
| smoking | 0.7881 0.7414 0.4383 0.0468 0.0000 0.0508 1.0000 | 0.7881 0.7874 0.4096 0.0007 0.0000 0.0085 1.0000 |
| KPSgroupI | 0.1610 0.1293 0.3358 0.0317 0.0000 0.0339 1.0000 | 0.1610 0.1547 0.3620 0.0064 0.0000 0.0254 1.0000 |
| KPSgroupII | 0.5085 0.4862 0.5002 0.0223 0.0000 0.0254 1.0000 | 0.5085 0.4986 0.5006 0.0099 0.0000 0.0085 1.0000 |
| KPSgroupIII | 0.3305 0.3845 0.4869 -0.0540 0.0000 0.0508 1.0000 | 0.3305 0.3468 0.4765 -0.0162 0.0000 0.0339 1.0000 |
| agegroup | 0.2966 0.2655 0.4420 0.0311 0.0000 0.0339 1.0000 | 0.2966 0.2952 0.4567 0.0014 0.0000 0.0085 1.0000 |
| BMgroupII | 0.2119 0.2500 0.4334 -0.0381 0.0000 0.0424 1.0000 | 0.2119 0.2020 0.4019 0.0099 0.0000 0.0000 0.0000 |
| BMgroupIII | 0.3814 0.3500 0.4774 0.0314 0.0000 0.0339 1.0000 | 0.3814 0.3962 0.4897 -0.0148 0.0000 0.0000 0.0000 |
| ECMGPA | 0.3814 0.3897 0.4881 -0.0083 0.0000 0.0085 1.0000 | 0.3814 0.3898 0.4883 -0.0085 0.0000 0.0085 1.0000 |
| BMTime | 0.7119 0.6931 0.4616 0.0188 0.0000 0.0169 1.0000 | 0.7119 0.7105 0.4541 0.0014 0.0000 0.0085 1.0000 |


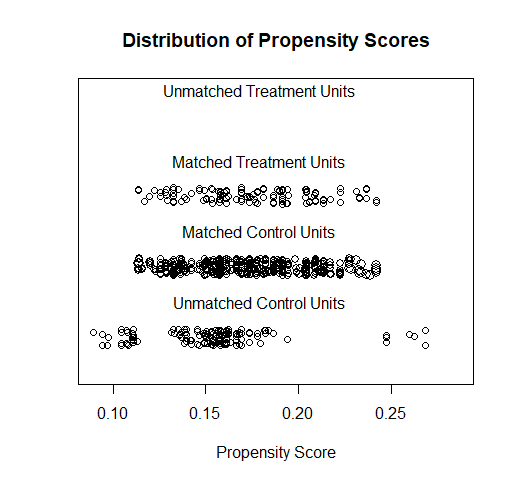

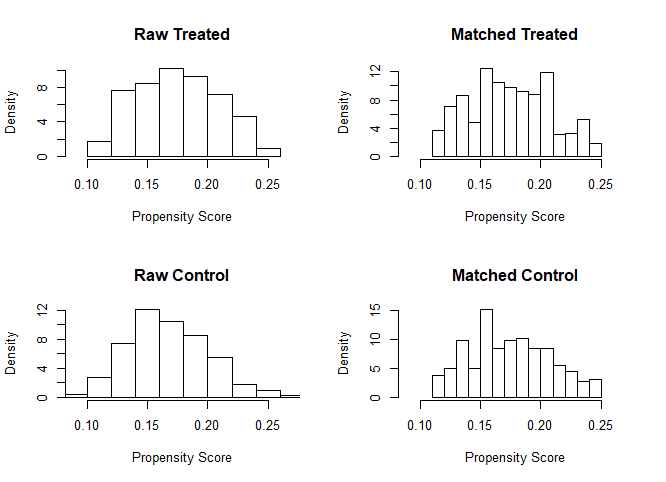


(2) Balance result of WBRT only (Group 1a) vs. BSC (Group 2) by PSM

| Group1a:Group2 | Summary of balance for all data(before PSM) 178:118 | Summary of balance for matched data(after PSM)106:106 |
| --- | --- | --- |
|  | Means Treated Means Control SD Control Mean Diff eQQ Med eQQ Mean eQQ Max | Means Treated Means Control SD Control Mean Diff eQQ Med eQQ Mean eQQ Max |
| distance | 0.4386 0.3721 0.1140 0.0665 0.0616 0.0682 0.1583 | 0.4136 0.4104 0.1141 0.0033 0.0019 0.0044 0.0242 |
| gender | 0.8390 0.7921 0.4069 0.0468 0.0000 0.0508 1.0000 | 0.8302 0.8113 0.3931 0.0189 0.0000 0.0189 1.0000 |
| smoking | 0.7881 0.6910 0.4634 0.0971 0.0000 0.1017 1.0000 | 0.7736 0.7547 0.4323 0.0189 0.0000 0.0189 1.0000 |
| KPSgroupI | 0.1610 0.0955 0.2947 0.0655 0.0000 0.0678 1.0000 | 0.1226 0.1321 0.3402 -0.0094 0.0000 0.0094 1.0000 |
| KPSgroupII | 0.5085 0.4944 0.5014 0.0141 0.0000 0.0169 1.0000 | 0.5189 0.5377 0.5009 -0.0189 0.0000 0.0189 1.0000 |
| KPSgroupIII | 0.3305 0.4101 0.4932 -0.0796 0.0000 0.0763 1.0000 | 0.3585 0.3302 0.4725 0.0283 0.0000 0.0283 1.0000 |
| agegroup | 0.2966 0.2809 0.4507 0.0157 0.0000 0.0169 1.0000 | 0.2453 0.2830 0.4526 -0.0377 0.0000 0.0377 1.0000 |
| BMgroupII | 0.2119 0.3034 0.4610 -0.0915 0.0000 0.0932 1.0000 | 0.2170 0.2547 0.4378 -0.0377 0.0000 0.0377 1.0000 |
| BMgroupIII | 0.3814 0.2978 0.4586 0.0836 0.0000 0.0847 1.0000 | 0.3491 0.3679 0.4845 -0.0189 0.0000 0.0189 1.0000 |
| ECMGPA | 0.3814 0.3764 0.4859 0.0050 0.0000 0.0085 1.0000 | 0.3774 0.3774 0.4870 0.0000 0.0000 0.0000 0.0000 |
| BMTime | 0.7119 0.8596 0.3484 -0.1477 0.0000 0.1441 1.0000 | 0.7925 0.7925 0.4075 0.0000 0.0000 0.0000 0.0000 |


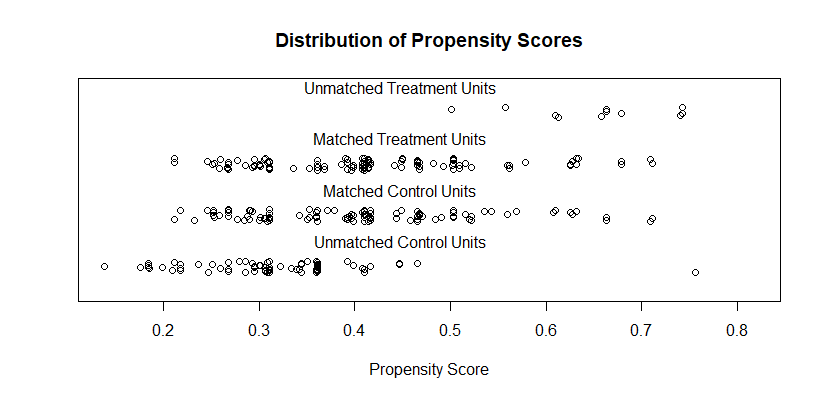

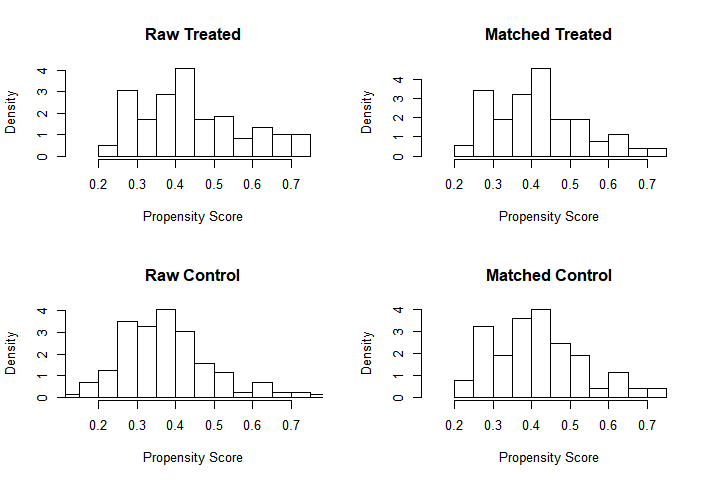


1. Balance result of Chemotherapy only (Group 1b) vs. BSC (Group 2) by PSM

| Group1b:Group2 | Summary of balance for all data(before PSM) 129:118 | Summary of balance for matched data(after PSM) 86:86 |
| --- | --- | --- |
|  | Means Treated Means Control SD Control Mean Diff eQQ Med eQQ Mean eQQ Max | Means Treated Means Control SD Control Mean Diff eQQ Med eQQ Mean eQQ Max |
| distance | 0.5406 0.4202 0.1771 0.1204 0.1234 0.1230 0.2701 | 0.5078 0.4976 0.1442 0.0102 0.0165 0.0172 0.0339 |
| gender | 0.8390 0.8295 0.3776 0.0095 0.0000 0.0169 1.0000 | 0.8372 0.8023 0.4006 0.0349 0.0000 0.0349 1.0000 |
| smoking | 0.7881 0.7907 0.4084 -0.0026 0.0000 0.0000 0.0000 | 0.8140 0.7442 0.4389 0.0698 0.0000 0.0698 1.0000 |
| KPSgroupI | 0.1610 0.1860 0.3907 -0.0250 0.0000 0.0254 1.0000 | 0.1977 0.1628 0.3713 0.0349 0.0000 0.0349 1.0000 |
| KPSgroupII | 0.5085 0.4961 0.5019 0.0124 0.0000 0.0169 1.0000 | 0.4302 0.5000 0.5029 -0.0698 0.0000 0.0698 1.0000 |
| KPSgroupIII | 0.3305 0.3178 0.4674 0.0127 0.0000 0.0169 1.0000 | 0.3721 0.3372 0.4755 0.0349 0.0000 0.0349 1.0000 |
| agegroup | 0.2966 0.2326 0.4241 0.0641 0.0000 0.0678 1.0000 | 0.2791 0.2907 0.4567 -0.0116 0.0000 0.0116 1.0000 |
| BMgroupII | 0.2119 0.1550 0.3634 0.0568 0.0000 0.0593 1.0000 | 0.2209 0.1744 0.3817 0.0465 0.0000 0.0465 1.0000 |
| BMgroupIII | 0.3814 0.4031 0.4924 -0.0217 0.0000 0.0169 1.0000 | 0.3837 0.4070 0.4942 -0.0233 0.0000 0.0233 1.0000 |
| ECMGPA | 0.3814 0.2636 0.4423 0.1178 0.0000 0.1186 1.0000 | 0.3953 0.3605 0.4830 0.0349 0.0000 0.0349 1.0000 |
| BMTime | 0.7119 0.4186 0.4953 0.2933 0.0000 0.2966 1.0000 | 0.6047 0.6047 0.4918 0.0000 0.0000 0.0000 0.0000 |


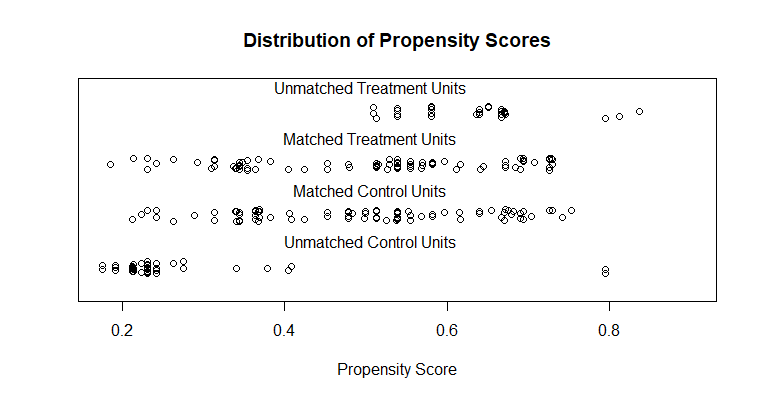

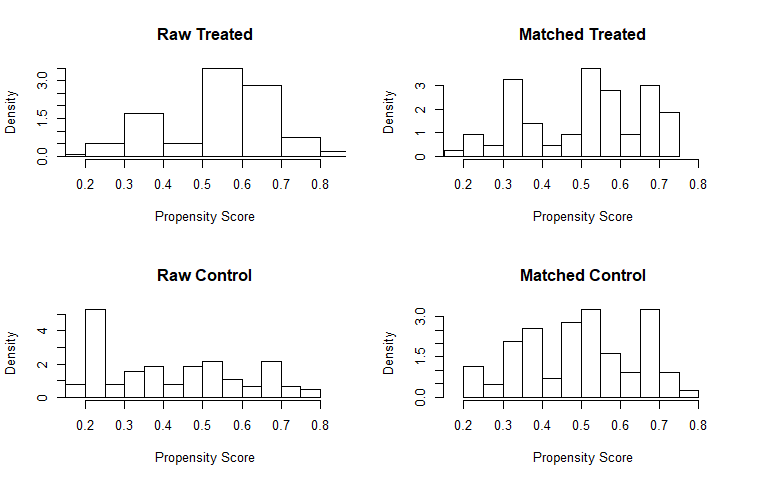


(4) Balance result of WBRT combined with Chemotherapy (Group 1c) vs. BSC (Group 2) by PSM

| Group1c:Group2 | Summary of balance for all data(before PSM) 273:118 | Summary of balance for matched data(after PSM) 114:114 |
| --- | --- | --- |
|  | Means Treated Means Control SD Control Mean Diff eQQ Med eQQ Mean eQQ Max | Means Treated Means Control SD Control Mean Diff eQQ Med eQQ Mean eQQ Max |
| distance | 0.3143 0.2964 0.0578 0.0179 0.0141 0.0188 0.0511 | 0.3110 0.3094 0.0618 0.0015 0.0013 0.0025 0.0103 |
| gender | 0.8390 0.8315 0.3750 0.0075 0.0000 0.0085 1.0000 | 0.8333 0.8246 0.3820 0.0088 0.0000 0.0088 1.0000 |
| smoking | 0.7881 0.7509 0.4333 0.0372 0.0000 0.0424 1.0000 | 0.7807 0.7456 0.4374 0.0351 0.0000 0.0351 1.0000 |
| KPSgroupI | 0.1610 0.1245 0.3308 0.0365 0.0000 0.0339 1.0000 | 0.1404 0.1491 0.3578 -0.0088 0.0000 0.0088 1.0000 |
| KPSgroupII | 0.5085 0.4762 0.5004 0.0323 0.0000 0.0339 1.0000 | 0.5175 0.4561 0.5003 0.0614 0.0000 0.0614 1.0000 |
| KPSgroupIII | 0.3305 0.3993 0.4906 -0.0688 0.0000 0.0678 1.0000 | 0.3421 0.3947 0.4910 -0.0526 0.0000 0.0526 1.0000 |
| agegroup | 0.2966 0.2711 0.4453 0.0255 0.0000 0.0254 1.0000 | 0.2982 0.2632 0.4423 0.0351 0.0000 0.0351 1.0000 |
| BMgroupII | 0.2119 0.2601 0.4395 -0.0482 0.0000 0.0508 1.0000 | 0.2193 0.2018 0.4031 0.0175 0.0000 0.0175 1.0000 |
| BMgroupIII | 0.3814 0.3590 0.4806 0.0224 0.0000 0.0254 1.0000 | 0.3860 0.4123 0.4944 -0.0263 0.0000 0.0263 1.0000 |
| ECMGPA | 0.3814 0.4579 0.4991 -0.0765 0.0000 0.0763 1.0000 | 0.3947 0.3596 0.4820 0.0351 0.0000 0.0351 1.0000 |
| BMTime | 0.7119 0.7143 0.4526 -0.0024 0.0000 0.0000 0.0000 | 0.7105 0.7807 0.4156 -0.0702 0.0000 0.0702 1.0000 |


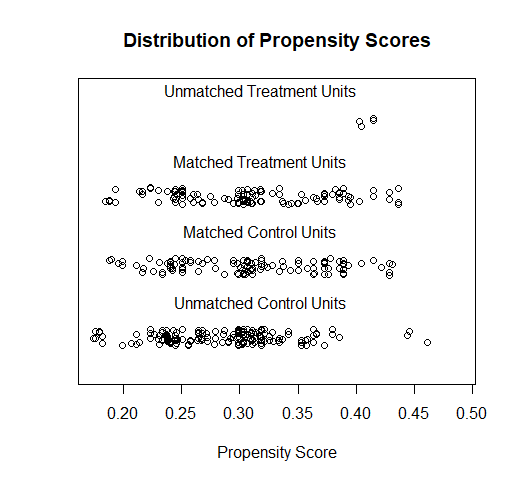

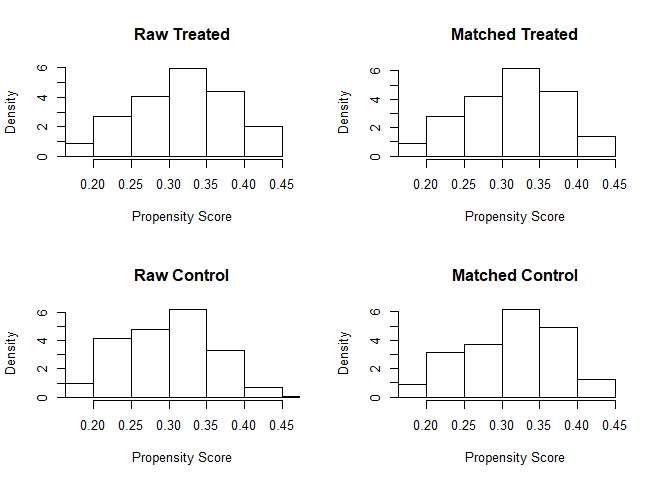

Supplement: Supplementary file 1 [file DataSheet_1.doc]
