# Supplementary material for: Best Supportive Care Versus Whole-Brain Irradiation, Chemotherapy Alone, or WBRT Plus Chemotherapy in Patients With Brain Metastases From Small-Cell Lung Cancer: A Case-Controlled Analysis
Source: Front Oncol. 2021 Mar 1;11:568568. doi: 10.3389/fonc.2021.568568 (PMC7957068; doi:10.3389/fonc.2021.568568)
Supplement: Supplementary file 2 [file DataSheet_2.doc]

Supplement 2. Univariate and Mutivariate analysis before and after matching

A: Univariate and Mutivariate analysis of 296 patients (Group 1a+ Group 2) before and after matching

| **Characteristics** | **Before PSM** | | | | | | | | **After PSM** | | | | | | | |
| --- | --- | --- | --- | --- | --- | --- | --- | --- | --- | --- | --- | --- | --- | --- | --- | --- |
|  | **Х^2^** | | **Log-rank** | | | **COX** | | | **Х^2^** | | **Log-rank** | | | **COX** | | |
|  | **Group 1a Group 2** | ***P*** | **MST**  **(m)** | **95%CI** | ***P*** | **HR** | **Z（Wald）** | ***P*** | **Group 1a Group 2** | ***P*** | **MST**  **(m)** | **95%CI** | ***P*** | **HR** | **Z（Wald）** | ***P*** |
| **Gender**  **Female**  **Male** | **37 19**  **141 99** | **0.314** | **8.5**  **5.0** | **6-17**  **3-7** | **0.002** | **1.456** | **1.601** | **0.109** | **20 18**  **86 88** | **0.72** | **7**  **5** | **5-12**  **3-7** | **0.1** | **—** | **—** | **—** |
| **Age**  **<65**  **≥65** | **128 83**  **50 35** | **0.770** | **6**  **5** | **5-8**  **3-7** | **0.08** | **1.238** | **1.541** | **0.123** | **76 80**  **30 26** | **0.533** | **6**  **5** | **4-8**  **4-8** | **0.2** | **—** | **—** | **—** |
| **Smoking**  **No**  **Yes** | **55 25**  **123 93** | **0.065** | **7**  **5** | **5-15**  **4-7** | **0.004** | **0.128** | **0.621** | **0.534** | **26 24**  **80 82** | **0.746** | **6.5**  **5.0** | **4-12**  **4-7** | **0.1** | **—** | **—** | **—** |
| **KPS**  **≤60**  **70-80**  **≥90** | **17 19**  **88 60**  **73 39** | **0.156** | **3.5**  **5.0**  **7.0** | **2-11**  **4-8**  **4-10** | **0.05** | **-0.1842** | **-0.937** | **0.349** | **14 13**  **57 55**  **35 38** | **0.907** | **4**  **5**  **7** | **2-11**  **4-7**  **4-10** | **0.2** | **—** | **—** | **—** |
| **Timing of BM metachronous**  **Synchronous** | **25 34**  **153 84** | **0.002** | **8**  **5** | **5-12**  **4-7** | **0.8** | **—** | **—** | **—** | **22 22**  **84 84** | **1.000** | **8.0**  **4.5** | **5-13**  **3-7** | **0.7** | **—** | **—** | **—** |
| **No. of BMs**  **1**  **2-3**  **>3** | **71 48**  **54 25**  **53 45** | **0.154** | **3**  **9**  **9** | **3-15**  **5-11**  **7-13** | **＜0.001** | **0.564**  **0.418** | **-3.624**  **-5.592** | **＜0.001**  **＜0.001** | **40 46**  **27 23**  **39 37** | **0.673** | **3.5**  **6.0**  **8.0** | **3-5**  **3-10**  **6-15** | **＜0.001** | **0.689**  **0.490** | **-1.973**  **-4.048** | **0.048** |
| **Extracranial**  **Disease Control**  **Yes**  **No** | **111 73**  **67 45** | **0.931** | **4**  **9** | **3-5**  **7-13** | **＜0.001** | **0.591** | **-3.889** | **＜0.001** | **66 66**  **40 40** | **1.000** | **4.0**  **7.5** | **3-6**  **6-12** | **＜0.001** | **0.601** | **-3.247** | **0.001** |
| **Treatment**  **Group 1a**  **Group 2** | **—** | **—** | **6.5**  **3.5** | **5-9**  **2-7** | **0.06** | **1.474** | **3.006** | **0.003** | **—** | **—** | **6.5**  **4.0** | **5-9**  **2-7** | **0.04** | **1.4007** | **2.292** | **0.22** |

MST: Medium Survival Time; SE: Stand Error; CI:Confidence Interval; HR: Hazard Ratio; Group 1a:WBRT only; Group 2:BSC;PSM: Propensity Score Matching; BM: Brain Metastases; KPS: Karnofsky

Performance Status score.

B: Univariate and Mutivariate analysis of 247 patients (Group 1b+ Group 2) before and after matching

| **Characteristics** | **Before PSM** | | | | | | | | **After PSM** | | | | | | | |
| --- | --- | --- | --- | --- | --- | --- | --- | --- | --- | --- | --- | --- | --- | --- | --- | --- |
|  | **Х^2^** | | **Log-rank** | | | **COX** | | | **Х^2^** | | **Log-rank** | | | **COX** | | |
|  | **Group 1b Group 2** | ***P*** | **MST**  **(m)** | **95%CI** | ***P*** | **HR** | **Z（Wald）** | ***P*** | **Group 1b Group 2** | ***P*** | **MST**  **(m)** | **95%CI** | ***P*** | **HR** | **Z（Wald）** | ***P*** |
| **Gender**  **Female**  **Male** | **22 19**  **107 99** | **0.841** | **7**  **5** | **6-12**  **4-7** | **0.1** | **—** | **—** | **—** | **17 14**  **69 72** | **0.552** | **7**  **4** | **6-12**  **3-7** | **0.08** | **1.170** | **0.419** | **0.6751** |
| **Age**  **<65**  **≥65** | **99 83**  **30 35** | **0.253** | **6**  **5** | **4-7**  **4-8** | **0.8** | **—** | **—** | **—** | **61 62**  **25 24** | **0.866** | **6**  **5** | **3-7**  **3-11** | **1** | **—** | **—** | **—** |
| **Smoking**  **No**  **Yes** | **27 25**  **102 93** | **0.961** | **7**  **5** | **5-12**  **4-7** | **0.09** | **1.3612** | **1.806** | **0.0709** | **22 16**  **64 70** | **0.270** | **7.5**  **4.0** | **6-13**  **3-7** | **0.02** | **1.4046** | **0.987** | **0.3235** |
| **KPS**  **≤60**  **70-80**  **≥90** | **24 19**  **64 60**  **41 39** | **0.873** | **3**  **5.5**  **7** | **2-10**  **4-7**  **5-10** | **0.06** | **0.8782**  **0.7410** | **-0.695**  **-1.450** | **0.4869**  **0.1471** | **14 17**  **43 37**  **29 32** | **0.642** | **2**  **6**  **7** | **1-11**  **4-7**  **4-10** | **0.2** | **—** | **—** | **—** |
| **Timing of BM metachronous**  **Synchronous** | **75 34**  **54 84** | **＜0.001** | **7**  **4** | **5-9**  **3-7** | **0.2** | **—** | **—** | **—** | **34 34**  **52 52** | **1.000** | **6.5**  **4.0** | **4-11**  **3-7** | **0.2** | **—** | **—** | **—** |
| **No. of BMs**  **1**  **2-3**  **>3** | **57 48**  **20 25**  **52 45** | **0.510** | **3**  **7**  **8** | **3-6**  **3-10**  **7-11** | **＜0.001** | **0.7004**  **0.5043** | **-1.869**  **-4.273** | **＜0.001** | **36 34**  **15 19**  **35 33** | **0.746** | **3**  **7**  **7** | **2-5**  **3-10**  **5-13** | **＜0.001** | **0.6232**  **0.4640** | **-2.117**  **-4.055** | **0.034**  **＜0.001** |
| **Extracranial**  **Disease Control**  **Yes**  **No** | **95 73**  **34 45** | **0.047** | **5**  **7** | **4-7**  **4-9** | **0.1** | **—** | **—** | **—** | **55 52**  **31 34** | **0.637** | **4**  **7** | **2-7**  **5-10** | **0.1** | **—** | **—** | **—** |
| **Treatment**  **Group 1b**  **Group 2** | **—** | **—** | **7.0**  **3.5** | **6-8**  **2-7** | **0.2** | **—** | **—** | **—** | **—** | **—** | **6.5**  **3.5** | **4-8**  **2-7** | **0.2** | **—** | **—** | **—** |

MST: Medium Survival Time; SE: Stand Error; CI:Confidence Interval; HR: Hazard Ratio; Group 1b:Chemotherapy only; Group 2:BSC;PSM: Propensity Score Matching; BM: Brain Metastases; KPS: Karnofsky

Performance Status score.

C: Univariate and Mutivariate analysis of 391 patients (Group 1c+ Group 2) before and after matching

| **Characteristics** | **Before PSM** | | | | | | | | **After PSM** | | | | | | | |
| --- | --- | --- | --- | --- | --- | --- | --- | --- | --- | --- | --- | --- | --- | --- | --- | --- |
|  | **Х^2^** | | **Log-rank** | | | **COX** | | | **Х^2^** | | **Log-rank** | | | **COX** | | |
|  | **Group 1c Group 2** | ***P*** | **MST**  **(m)** | **95%CI** | ***P*** | **HR** | **Z（Wald）** | ***P*** | **Group 1c Group 2** | ***P*** | **MST**  **(m)** | **95%CI** | ***P*** | **HR** | **Z（Wald）** | ***P*** |
| **Gender**  **Female**  **Male** | **46 19**  **227 99** | **0.855** | **11**  **11** | **9-16**  **9-13** | **0.6** | **—** | **—** | **—** | **20 19**  **94 95** | **0.860** | **11**  **10** | **7-17**  **7-12** | **0.3** | **—** | **—** | **—** |
| **Age**  **<65**  **≥65** | **199 83**  **74 35** | **0.605** | **11**  **10** | **10-14**  **8-13** | **0.05** | **1.154** | **1.151** | **0.250** | **84 80**  **30 34** | **0.555** | **10**  **8** | **8-12**  **5-13** | **0.4** | **—** | **—** | **—** |
| **Smoking**  **No**  **Yes** | **68 25**  **205 93** | **0.427** | **11**  **11** | **9-16**  **9-13** | **0.3** | **—** | **—** | **—** | **29 25**  **85 89** | **0.533** | **11**  **9** | **8-17**  **7-12** | **0.1** | **—** | **—** | **—** |
| **KPS**  **≤60**  **70-80**  **≥90** | **34 19**  **130 60**  **109 39** | **0.365** | **6**  **10**  **13** | **4-12**  **9-13**  **11-17** | **＜0.001** | **0.755**  **0.510** | **-1.623**  **-3.662** | **0.105**  **＜0.001** | **17 16**  **52 59**  **45 39** | **0.638** | **9**  **9**  **13** | **3-14**  **7-12**  **7-18** | **0.01** | **0.9028** | **-0.464** | **0.6424**  **0.0376** |
| **Timing of BM metachronous**  **Synchronous** | **78 34**  **195 84** | **0.961** | **10**  **11** | **8-13**  **10-13** | **0.03** | **0.876** | **-1.062** | **0.288** | **25 33**  **89 81** | **0.224** | **11**  **10** | **8-14**  **7-12** | **0.3** | **—** | **—** | **—** |
| **No. of BMs**  **1**  **2-3**  **>3** | **104 48**  **71 25**  **98 45** | **0.596** | **8**  **12**  **15** | **6-10**  **10-16**  **13-17** | **＜0.001** | **0.715**  **0.504** | **-2.319**  **-5.251** | **0.020** | **44 45**  **23 25**  **47 44** | **0.908** | **6**  **10**  **16** | **4-9**  **7-14**  **11-20** | **＜0.001** | **0.7676**  **0.4041** | **-1.345**  **-5.288** | **0.1788**  **＜0.001** |
| **Extracranial**  **Disease Control**  **Yes**  **No** | **148 73**  **125 45** | **0.161** | **10**  **13** | **8-12**  **10-15** | **0.07** | **0.826** | **-1.704** | **0.088** | **73 69**  **41 45** | **0.585** | **8**  **12** | **6-11**  **9-15** | **0.02** | **0.7379** | **-2.010** | **0.044** |
| **Treatment**  **Group 1c**  **Group 2** | **—** | **—** | **14.0**  **3.5** | **13-16**  **2-7** | **＜0.001** | **2.428** | **7.286** | **＜0.001** | **—** | **—** | **14**  **4** | **12-16**  **2-7** | **＜0.001** | **2.5343** | **6.242** | **＜0.001** |

MST: Medium Survival Time; SE: Stand Error; CI:Confidence Interval; HR: Hazard Ratio; Group 1c:WBRT+Chemotherapy, Group 2:BSC;PSM: Propensity Score Matching; BM: Brain Metastases; KPS: Karnofsky

Performance Status score.
